# Hermaphroditism promotes mate diversity in flowering plants

**DOI:** 10.1002/ajb2.1336

**Published:** 2019-08-12

**Authors:** Dorothy A. Christopher, Randall J. Mitchell, Dorset W. Trapnell, Patrick A. Smallwood, Wendy R. Semski, Jeffrey D. Karron

**Affiliations:** ^1^ Department of Biological Sciences University of Wisconsin – Milwaukee 3209 N. Maryland Ave Milwaukee Wisconsin 53211 USA; ^2^ Department of Biology University of Akron Akron Ohio 44325 USA; ^3^ Department of Plant Biology University of Georgia 120 Carlton St Athens Georgia 30602 USA

**Keywords:** hermaphrodite, male fitness, mate diversity, mating network, mating portfolio, multiple paternity, paternity, pollination, selfing, sexual system

## Abstract

**Premise:**

Genetically diverse sibships are thought to increase parental fitness through a reduction in the intensity of sib competition, and through increased opportunities for seedling establishment in spatially or temporally heterogeneous environments. Nearly all research on mate diversity in flowering plants has focused on the number of fathers siring seeds within a fruit or on a maternal plant. Yet as hermaphrodites, plants can also accrue mate diversity by siring offspring on several pollen recipients in a population. Here we explore whether mate composition overlaps between the dual sex functions, and discuss the implications for plant reproductive success.

**Methods:**

We established an experimental population of 49 *Mimulus ringens* (monkeyflower) plants, each trimmed to a single flower. Following pollination by wild bees, we quantified mate composition for each flower through both paternal and maternal function. Parentage was successfully assigned to 240 progeny, 98% of the sampled seeds.

**Results:**

Comparison of mate composition between male and female function revealed high mate diversity, with almost no outcross mates shared between the two sexual functions of the same flower.

**Conclusions:**

Dual sex roles contribute to a near doubling of mate diversity in our experimental population of *Mimulus ringens*. This finding may help explain the maintenance of hermaphroditism under conditions that would otherwise favor the evolution of separate sexes.

Most flowering plants are hermaphrodites, and therefore accrue fitness both through pollen export (male function) and pollen receipt (female function). Several potential advantages of these dual sex functions have been proposed, including reproductive assurance, efficient pollinator attraction, and the ability to optimize resource allocation to maximize reproductive success over the flowering season (Charnov et al., 1976; Lloyd, 1982). Although previous work has focused on the number of offspring produced through male and female function (e.g., Briscoe Runquist et al., 2017), the parental diversity of those offspring is rarely quantified but may be of great importance (Barrett and Harder, 2017). Here we show that hermaphroditism nearly doubles mate diversity, and discuss the implications for evolutionary processes in flowering plant populations.

Flowering plants are sessile and cannot directly control pollen dispersal and pollen receipt (Harper, 1977). Since foraging pollinators often move short distances between neighboring plants, and seed dispersal is frequently limited, individuals are often surrounded by close relatives, generating spatial genetic structure (Turner et al., 1982; Vekemans and Hardy, 2004), and limiting the diversity of accessible mates. One way that plants can increase mate diversity is by participating in multiple mating events, for example by receiving visits from several pollinators with different foraging itineraries (Karron et al., 2006). This increases the likelihood of mating between unrelated plants, lessening the incidence of biparental inbreeding and potential for inbreeding depression (Pannell and Labouche, 2013). Additionally, when genetic diversity within a sibship is increased, it may reduce the intensity of local competition among siblings (Karron and Marshall, 1990, 1993) and therefore may increase parental fitness. Increased mate diversity also produces increased variance in offspring fitness, potentially generating novel genotypes that may be successful in spatially or temporally heterogeneous environments (Williams, 1975).

Nearly all research on mate diversity in flowering plants has focused on the number of fathers siring seeds within a fruit or on a maternal plant (Bernasconi, 2003; Karron et al., 2006; Pannell and Labouche, 2013; Krauss et al., 2017). Yet as hermaphrodites, plants can also increase mate diversity by siring offspring on several pollen recipients in the population. Characterizing “mating portfolios” would provide a powerful way to analyze mate diversity through both sex functions, but data from the paternal perspective are scarce (Garcia‐Gonzalez et al., 2015; Barrett and Harder, 2017). Mating portfolios that contain only the maternal perspective are likely to underestimate mate diversity; however, the extent of this underestimation depends on the similarity in mate composition from the paternal and maternal perspectives. That similarity has never before been measured. Characterization of mating portfolios requires detailed information about parentage of paternal and maternal sibships in a population. Here we use this approach to quantify mate composition through maternal and paternal function in an experimental population of monkeyflower.

## MATERIALS AND METHODS

### Study system

*Mimulus ringens* L. (Phrymaceae) is a diploid, self‐compatible wetland plant native to central and eastern North America. The showy purple flowers last for just a single morning, and are pollinated primarily by bumblebees (*Bombus* spp.) (Mitchell et al., 2004). The anthers dehisce before dawn, and individual flowers typically receive 1‐3 visits by bumblebee workers before stigmas close in late morning (Mitchell et al., 2005). Nearly all flowers produce capsules with numerous seeds (Karron and Mitchell, 2012). The mean number of seeds per capsule in the present study was 2483 (± 511). Pollinator‐mediated gene dispersal is extremely limited in *M. ringens* and most pollen from donor flowers only moves to the next three flowers in the visitation sequence (Holmquist et al., 2012).

### Experimental design

As part of a larger study to examine how competition for pollination affects mating patterns, we constructed an experimental array of 49 unique genets spaced 0.8 m apart in a square grid. These experimental plants were grown from seed collected from a single natural population in the Panzner Wetland Wildlife Reserve near Akron, Ohio, USA (41.068524**°**N, 81.612118**°**W). Plants in this population typically have displays with 1‐2 flowers per day, and 6‐8 flowers over the season. Plants were grown in 20 cm pots in a common garden at the University of Wisconsin‐Milwaukee Field Station (Saukville, Wisconsin, USA; 43.387335**°**N, 88.022870**°**W), and all plants flowered simultaneously. The environmental conditions were homogenous across the array, i.e., all plants were in full sun and were watered equally every day. The experimental array was surrounded by a restored wet meadow with a high diversity of bumblebee‐pollinated species. No other *Mimulus* plants occurred within 100 m of the experimental array. On 25 July 2017, plants were trimmed to a single flower before anthers dehisced and before pollinators became active, at approximately 0530h. Pollinators were allowed to forage naturally through the array. We tagged flowers at 1300h, several hours after all stigmas had closed. All 49 flowers produced fruits, which we collected on 28 August 2017.

### Data analyses

We genotyped 5 seedlings per fruit for paternity assignment. This sample size was selected because effective mate number in a previous study of *Mimulus ringens* was 3.97 ± 0.60 (Mitchell et al., 2005, 2013) and the highly restricted pollen carryover found in *M. ringens* minimizes mate diversity within fruits (Holmquist et al., 2012). We genotyped the progeny arrays as well as the 49 parental individuals at eight microsatellite loci following Nunziata et al. (2012) (Appendices [Link], [Link]). All eight loci were trinucleotide repeats. The mean pairwise relatedness among the 49 parental individuals was –0.010 (SE = 0.003). The multilocus exclusion probability for paternity assignment when the maternal individual was known was 0.98. The final dataset had 1% missing data. Null alleles were rare in the progeny at all 8 loci (mean frequency 0.002; range 0.000 to 0.004).

We performed the paternity analysis on all 245 seedlings using Cervus 3.0 (Kalinowski et al., 2007). Cervus uses a maximum likelihood procedure to identify the most likely father. In the analysis we retained the default 2% error rate, and we included the known maternal genotype. Paternity was assigned to 240 seedlings. Forty‐seven percent of the 240 seedlings were assigned with >95% confidence; 53% of the seedlings were assigned with confidence between 80 and 94%. Of the 240 seedlings, 36 were selfed (15% selfing rate). Paternity was unresolved—a single father could not be identified at 80% confidence or above—for 5 seedlings, and thus they were excluded from analyses.

For each parental plant, we identified matings from both maternal and paternal perspectives; i.e., we identified the maternal plants on which each individual sired seeds, and we identified the fathers of seeds mothered by each plant. With these data, we were able to compare mate diversity acquired through siring seeds on multiple maternal plants as well as through mothering seeds fathered by multiple individuals. We also calculated the similarity of outcross mate composition between paternal and maternal function for each individual using the Jaccard index. The Jaccard index quantifies the similarity of two sets of data. It varies between 0 and 1, with larger values indicating higher similarity between the two sets. Mating portfolios were visualized using the ‘bipartiteD3’ package (Terry, 2018) in R version 3.5.2 (R Core Team, 2018).

We also assessed whether there was a spatial effect on male or female fertility. We used an ANOVA to determine if there was a significant difference between the number of seeds sired by plants along the perimeter of the population vs. in the interior. We did the same for the number of sires per fruit.

## RESULTS

**Figure 1:**
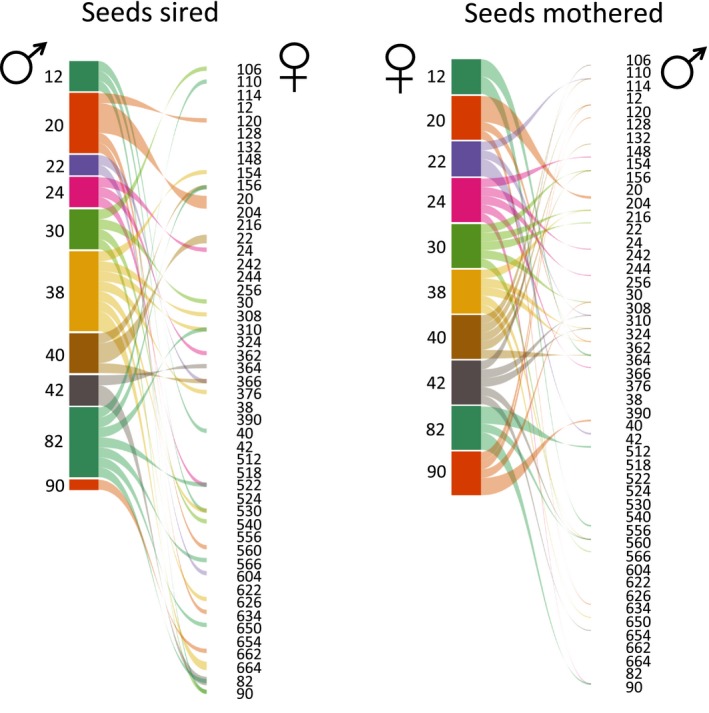
Mating portfolios for 10 focal plants with random positions in an experimental population of 49 plants. Note that all 49 individuals are included as potential pollen recipients (left panel) or pollen donors (right panel). The links between them show that the father on the left sired seeds on the individual labeled on the right. The thickness of the link is scaled to the number of seeds sired. The height of the colored bars (left) for each individual is scaled to show the proportion of that individual's contribution to the sum of the number of seeds sired for the 10 individuals. The “seeds mothered” (right panel) depicts the maternal individual (left) and the individuals who sired seeds on the maternal plant are linked on the right. The thickness of the link is scaled to the number of seeds from one fruit that were sired by the paternal individual. The height of the colored bar (left) for each individual is scaled to the proportion of the total number of seeds that were mothered by each individual.

All individuals acquired fitness through both male and female function; that is, they both served as maternal plants and sired seeds on other individuals. Mate composition from the male and female perspective differed dramatically for most plants (Fig. 1). Overall in the population the mean Jaccard similarity between outcross male and female function was 0.06 (range = 0–0.18, N = 49), a very low level of overlap between sexual functions. The mean number of mates per mother (sires per fruit) was 3.8 (SE = 0.40). The mean number of mates for each pollen donor was 4.4 (SE = 0.35).

There was no significant effect of spatial position in the array on the number of seeds sired (*F*_1,47_ = 2.11, *p* = 0.15). Plants around the edge of the array (N = 24) sired a mean of 5.05 (SE = 0.42) seeds, while plants in the interior of the array (N = 25) sired a mean of 4.0 (SE = 0.28) seeds. Similarly, there was no effect of spatial position on the number of sires per fruit (*F*_1,47_ = 0.39, *p* = 0.85). Plants around the edge of the array (N = 24) had a mean of 3.7 (SE = 0.54) sires per fruit, while plants in the interior of the array (N = 25) had a mean of 3.8 (SE = 0.51) sires per fruit.

**Figure 2:**
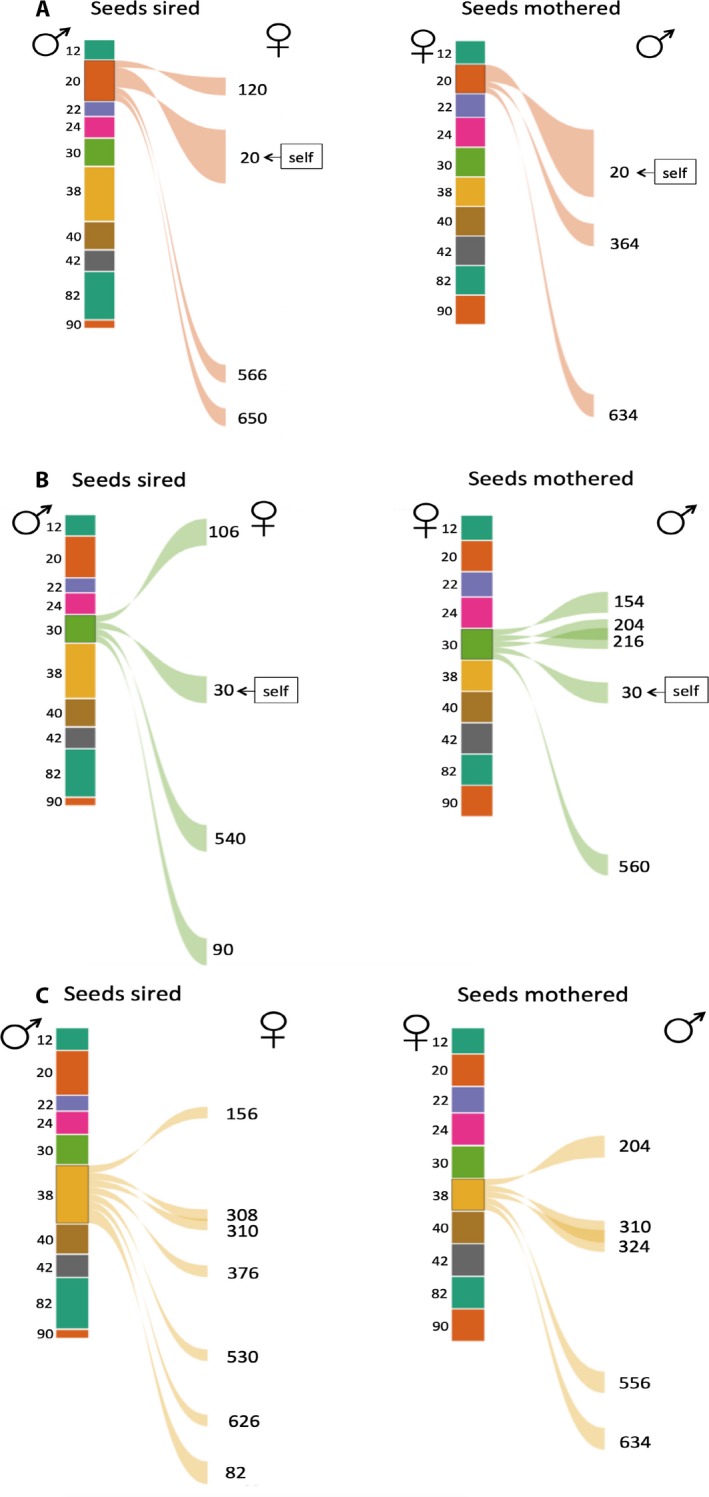
Detailed mating portfolios for 3 of the 10 focal plants shown in Figure 1. The thickness of the link is scaled to the number of seeds sired (left panel) or mothered (right panel). (A) The matings for plant 20 from the male (left panel) and female (right panel) perspectives; (B) the matings for plant 30; and (C) the matings for plant 38.

Three individuals from the population highlight the patterns seen in the full dataset (Fig. 2). The Jaccard similarity of outcross mate composition in plants 20 and 30 was 0; in both plants, there were no outcross mates shared between male and female function on the same plant. The Jaccard similarity between outcross male and female function in plant 38 was 0.09; plant 310 served as a pollen donor and pollen recipient of plant 38.

Plant 20 sired outcross seeds on three plants and also sired seeds through self‐pollination (Fig. 2A, left panel). Plant 20 mothered seeds that were sired by two outcross pollen donors and mothered seeds through self‐pollination (Fig. 2A, right panel). Plant 30 (Fig. 2B, left panel) sired seeds on three outcross plants and also sired one seed through self‐pollination. Plant 30 mothered seeds that were sired by four outcross pollen donors and as well as one seed through self‐pollination (Fig. 2B, right panel). Plant 38 sired outcross seeds on seven plants (Fig. 2C, left panel) and mothered outcross seeds sired by five pollen donors (Fig. 2C, right panel). Plant 38 did not produce selfed seeds.

## DISCUSSION

Comparison of mate composition between male and female function revealed high mate diversity, with almost no outcross mates shared between the two sexual functions of the same plant. The importance of the paternal contribution to fitness through seed quality has often been neglected; here we show that dual sex functions can nearly double mate diversity in hermaphroditic plants. Because environments are often spatially heterogeneous and seed dispersal is limited, greater variance in the genetic diversity of maternal half‐sibs increases the likelihood that some progeny will be well suited for their particular microhabitat. Additionally, because paternal half‐sibs are more widely dispersed than maternal half‐sibs, competition decreases between related individuals.

Our study highlights differences in the male and female mate composition of individual flowers. We hypothesize that when plants produce several flowers over multiple days, or have many visits per flower, differences between paternal and maternal mating portfolios at the plant level are likely to be diminished due to increased sampling of donors through both sexual functions. Therefore, the mate diversity advantages associated with hermaphroditism may be most pronounced in plants that produce few flowers or have low visitation.

It is important to note that we sampled a relatively small proportion of the total seeds produced per fruit. It is possible that sampling more seeds would have revealed additional sires per fruit, and thus our results may slightly underestimate the overlap between male and female function. However, because pollen carryover is very limited in *Mimulus ringens* (Holmquist et al., 2012), it is likely that most of the mates have been sampled.

Plants commonly experience heterogeneous pollination environments due to the foraging patterns of their pollinators. Thus, the first plants visited on a foraging bout may acquire more of their fitness and mate diversity through male outcross function, while plants at the end of a pollinator's foraging bout may acquire more of their fitness and mate diversity through female function. In these conditions conclusions about the mate diversity of hermaphroditic plants based only on female function might be fundamentally biased, since plants with low diversity through female function might often have high diversity through male function.

Male and female mate composition is also likely to be influenced by differences among pollinators in foraging behavior and grooming (Rhodes et al., 2017; Weber, 2017; Minnaar et al., 2019; Valverde et al., 2019). We expect that species pollinated by traplining pollinators will have low similarity in male and female mate composition because traplining pollinators (e.g., bumblebees) often visit plants in repeatable sequences and do not return to previously sampled flowers during one foraging bout (Ohashi and Thomson, 2009). By contrast, species pollinated by territorial pollinators that may approach a focal plant from several neighbors are likely to have higher overlap in male and female mate composition (Krauss et al., 2017).

Additionally, population size may have an effect on mate diversity. Mate composition between the two sex functions may be more similar in small than in large populations. For example, bumblebees are more likely to revisit plants in small populations, leading to overlap between the donors visited prior to a focal flower and recipients visited after a focal flower. By contrast, as population size increases, the likelihood of revisitation diminishes, reducing overlap in mate composition between the two sexual functions.

Because plants gain fitness through male and female function, considering both sex functions is critical to understanding the evolutionary trajectory of populations. In particular, estimating mate diversity from both perspectives is necessary to examine intra‐population gene movement (Fortuna et al., 2008). Differences in the sizes of maternal seed shadows and pollen clouds from different donors (Cook, 1980) suggest that information may be lost if gene dispersal is assessed based only on one sex function.

The mating portfolio approach may have useful applications to hermaphroditic animal systems, which represent 5–6% of all animal species (Jarne and Auld, 2006). These animals may experience pronounced differences in mate diversity between male vs. female function due to high variability in mating assortment (Pélissié et al., 2012; Janicke et al., 2013; McDonald and Pizzari, 2018), and mating portfolios may help elucidate mating network patterns in hermaphroditic animals.

Finally, many species with unisexual individuals are thought to evolve from a hermaphroditic ancestor (Charlesworth and Charlesworth, 1978; Renner, 2014). Obligate outcrossing and a reduction in inbreeding depression relative to hermaphroditism are key advantages that favor the evolution of separate sexes (Dufay and Billard, 2012). However, increased mate diversity may provide an advantage to hermaphroditism, and may contribute to the maintenance of hermaphroditism under conditions that would otherwise favor separate sexes (e.g., harsh biotic or abiotic conditions; Ashman, 2002; Vaughton and Ramsey, 2012). Additional research is needed to compare mate diversity in species with hermaphrodite and unisexual individuals to further our understanding of how sexual systems evolve.

## Supporting information


**APPENDIX S1.** Table of microsatellite allele frequencies.

**APPENDIX S2.** Table of exclusion probabilities by microsatellite locus.

## Data Availability

Data available from the Dryad Digital Repository: https://doi.org/10.5061/dryad.r7565db (Christopher et al., 2019).
