# Supplementary material for: Hermaphroditism promotes mate diversity in flowering plants
Source: Am J Bot. 2019 Aug 12;106(8):1131–6. doi: 10.1002/ajb2.1336 (PMC6852098; doi:10.1002/ajb2.1336)
Supplement: Supplementary file 1 — APPENDIX S1. Table of microsatellite allele frequencies. [file AJB2-106-1131-s001.docx]

**Appendix S1.** Table of microsatellite allele frequencies.

Allele frequencies of the parental individuals are shown for each of the eight loci used in this study. (For more information about these loci, see Nunziata et al. 2012).

***Locus Allele Frequency Locus Allele Frequency***

**MIRI24** 235 0.01 **MIRI82** 162 0.31

244 0.03 168 0.04

256 0.07 171 0.38

259 0.34 174 0.28

262 0.11 **MIRI85** 139 0.32

265 0.12 142 0.20

268 0.24 145 0.48

271 0.08 **MIRI92**  307 0.04

**MIRI27** 151 0.48 310 0.02

154 0.03 319 0.24

160 0.24 322 0.70

179 0.21 **MIRI95** 240 0.02

182 0.03 243 0.22

**MIRI46** 235 0.03 246 0.05

244 0.03 249 0.02

247 0.02 252 0.19

256 0.05 255 0.49

259 0.03

263 0.69

266 0.07

269 0.07

**MIRI58** 157 0.14

160 0.53

163 0.17

166 0.01

169 0.07

172 0.07
