# Supplementary material for: Hermaphroditism promotes mate diversity in flowering plants
Source: Am J Bot. 2019 Aug 12;106(8):1131–6. doi: 10.1002/ajb2.1336 (PMC6852098; doi:10.1002/ajb2.1336)
Supplement: Supplementary file 2 — APPENDIX S2. Table of exclusion probabilities by microsatellite locus. [file AJB2-106-1131-s002.docx]

**Appendix S2.** Table of exclusion probabilities by microsatellite locus*.*

The table below shows the single locus exclusion probabilities for paternity assignment with a known mother.

***Locus Single locus***

***exclusion probability***

**MIRI24** 0.60

**MIRI27** 0.40

**MIRI46** 0.33

**MIRI58** 0.44

**MIRI82** 0.41

**MIRI85** 0.34

**MIRI92** 0.22

**MIRI95** 0.66
